# Bis[(2-methyl­benz­yl)bis­(pyridin-2-ylmethyl-κ*N*)amine-κ*N*]manganese(II) bis­(perchlorate)

**DOI:** 10.1107/S1600536814003055

**Published:** 2014-02-15

**Authors:** Ray J. Butcher, Yilma Gultneh, T. B. Yisgedu

**Affiliations:** a Department of Chemistry, Howard University, 525 College Street NW, Washington, DC 20059, USA

## Abstract

In the title complex, [Mn(C_20_H_21_N_3_)_2_](ClO_4_)_2_, two tridentate (2-methyl­benz­yl)bis­(pyridin-2-ylmeth­yl)amine (*L*) ligands form the Mn^II^ complex [Mn*L*_2_](ClO_4_)_2_. The Mn^II^ ion lies on a twofold axis and the complex cation is significantly distorted from regular octa­hedral geometry. The packing is stabilized by weak C—H⋯O inter­actions between the cations and anions, which link them into a zigzag ribbon along [101]. The perchlorate anion is disordered and was constrained to be tetra­hedral with two orientations having occupancies of 0.768 (4) and 0.232 (4). The 2-methylbenzyl moiety is also disordered over two sets of sites, with occupancies of 0.508 (15) and 0.492 (15).

## Related literature   

For the importance of flexible coordination complexes of Mn in biomimetic chemistry, see: Zhou *et al.* (2011 ▶); Walsdorff *et al.* (1999 ▶); Nielsen *et al.* (2007 ▶); Routasalo *et al.* (2008 ▶), in catalysis, see: Raycroft *et al.* (2012 ▶); Berthet *et al.* (2013 ▶), in medicinal chemistry, see: Ari *et al.* (2013 ▶); Chang *et al.* (2004 ▶), in O_2_ activation and catalysis of redox reactions and oxygenation of organic substrates, see: Karlin *et al.* (1984 ▶); Karlin & Gultneh (1987 ▶); Hatcher & Karlin (2004 ▶) and in making polymeric materials that form by self-assembling metal coord­ination compounds, see: Denmark & Jacobsen (2000 ▶); Chatterjee (2008 ▶); Katsuki (2004 ▶); Kim *et al.* (2010 ▶). For the study of active sites of enzymes in biological systems as well as in synthetic complexes of inter­est, see: Davies *et al.* (2004 ▶). For the preparation of bis­(pyridin-2-ylmeth­yl)amine (bpa), see: Romary *et al.* (1967 ▶). For structures of similar Mn complexes, see: Glerup *et al.* (1992 ▶); Gultneh *et al.* (2006 ▶).
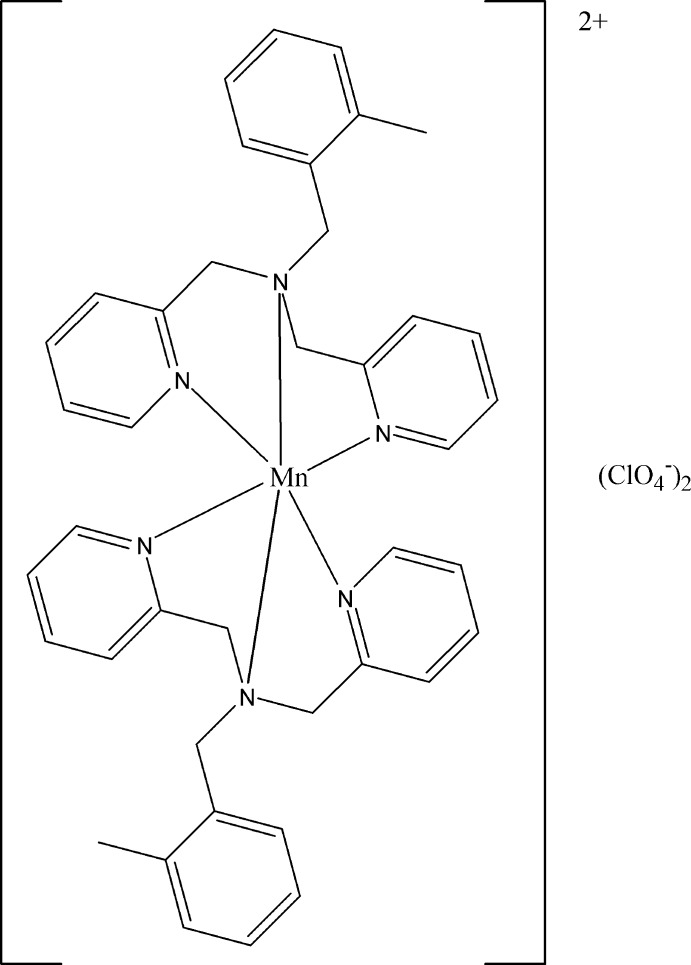


## Experimental   

### 

#### Crystal data  

[Mn(C_20_H_21_N_3_)_2_](ClO_4_)_2_
*M* *_r_* = 860.63
Monoclinic, 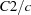

*a* = 23.162 (3) Å
*b* = 10.4755 (11) Å
*c* = 19.391 (2) Å
β = 118.896 (8)°
*V* = 4119.1 (9) Å^3^
*Z* = 4
Mo *K*α radiation
μ = 0.51 mm^−1^
*T* = 293 K
0.42 × 0.37 × 0.18 mm

#### Data collection  

Bruker P4 diffractometer
Absorption correction: empirical (using intensity measurements) (*XEMP*; Siemens, 1989 ▶) *T*_min_ = 0.72, *T*_max_ = 0.92
4863 measured reflections
4750 independent reflections
3075 reflections with *I* > 2σ(*I*)
*R*_int_ = 0.021
3 standard reflections every 97 reflections intensity decay: none

#### Refinement  

*R*[*F* ^2^ > 2σ(*F* ^2^)] = 0.065
*wR*(*F* ^2^) = 0.193
*S* = 1.03
4750 reflections
299 parameters
86 restraints
H-atom parameters constrained
Δρ_max_ = 0.67 e Å^−3^
Δρ_min_ = −0.44 e Å^−3^

### 

Data collection: *XSCANS* (Siemens, 1991 ▶); cell refinement: *XSCANS*; data reduction: *XDISK* (Siemens, 1991 ▶); program(s) used to solve structure: *SHELXS97* (Sheldrick, 2008 ▶); program(s) used to refine structure: *SHELXL2013* (Sheldrick, 2008 ▶); molecular graphics: *SHELXTL* (Sheldrick, 2008 ▶); software used to prepare material for publication: *SHELXTL*.

## Supplementary Material

Crystal structure: contains datablock(s) I. DOI: 10.1107/S1600536814003055/mw2117sup1.cif

Structure factors: contains datablock(s) I. DOI: 10.1107/S1600536814003055/mw2117Isup2.hkl

CCDC reference: 986203

Additional supporting information:  crystallographic information; 3D view; checkCIF report

## Figures and Tables

**Table 1:** Hydrogen-bond geometry (Å, °)

*D*—H⋯*A*	*D*—H	H⋯*A*	*D*⋯*A*	*D*—H⋯*A*
C3*A*—H3*AA*⋯O3*A* ^i^	0.93	2.51	3.106 (5)	122
C6*A*—H6*AA*⋯O1*A* ^ii^	0.93	2.57	3.420 (10)	152
C6*A*—H6*AA*⋯O2*A* ^ii^	0.93	2.56	3.309 (12)	138
C1*B*—H1*BB*⋯O4	0.97	2.49	3.266 (5)	136
C1*B*—H1*BB*⋯O1*A*	0.97	2.54	3.356 (11)	142
C3*B*—H3*BA*⋯O1	0.93	2.47	3.300 (6)	149

Symmetry codes: (i) 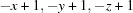; (ii) 
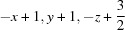
.

**Fig. 1.**
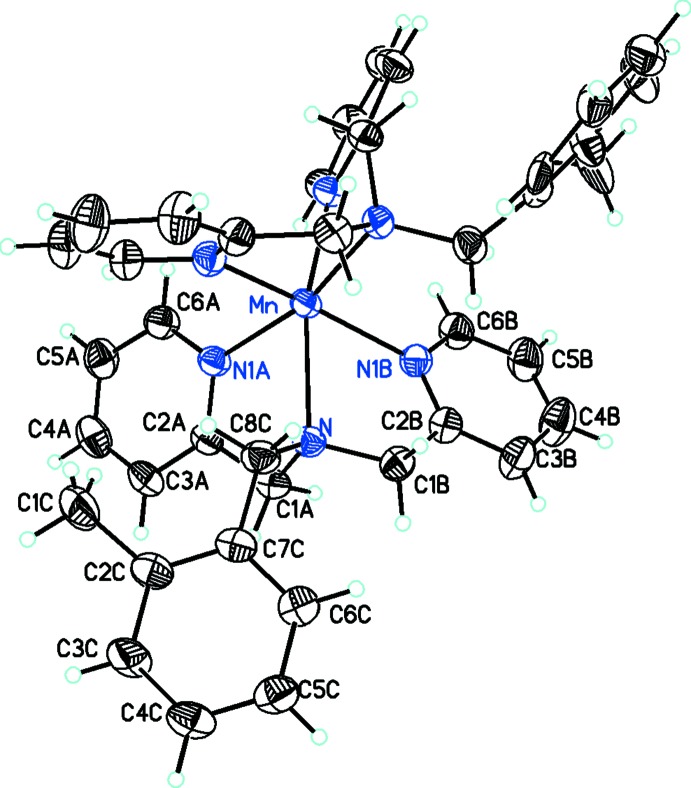
ORTEP diagram of the complex cation showing the atom numbering scheme for the unique portion.

**Fig. 2.**
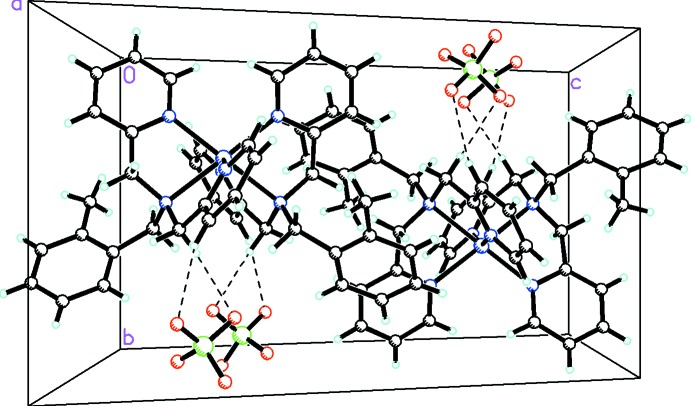
Packing diagram for the complex viewed along the a axis. C—H···O interactions are shown by dashed lines.

## supplementary crystallographic information

### 1. Comment

The chelating ligands, bis(pyridin-2-ylalky)amine (tridentate) and tris (pyridin-2-ylalkyl) amine (tetradentate) (alkyl = methyl or ethyl) form coordination complexes with a variety of transition metals, including Cu, Fe, and Mn, with variable oxidation states and with a high degree of flexibility and stability. Such complexes are important in biomimetic coordination chemistry (Zhou, *et al.*, 2011; Walsdorff, *et al.*, 1999; Nielsen, *et al.*, 2007; Routasalo, *et al.*, 2008), catalysis (Raycroft, *et al.*, 2012; Berthet *et al.*, 2013), medicinal chemistry (Ari *et al.*, 2013: Chang, *et al.*, 2004), O_2_ activation, catalysis of redox reactions and oxygenation of organic substrates (Karlin, *et al.*, 1984; Karlin & Gultneh, 1987; Hatcher & Karlin, 2004), and making polymeric materials that form by self-assembling metal coordination compounds (Denmark & Jacobsen, 2000; Chatterjee, 2008; Katsuki, 2004; Kim, *et al.*, 2010). Studies of the coordination, structural and reactivity features of this class of ligands with various metal ions have become an important tool in understanding the detailed structures and reaction mechanisms at the active sites of enzymes in biological systems as well as in synthetic complexes of interest (Davies *et al.*, 2004).

In the title complex, two linear, chelating tridentate ligand molecules (*L*) form a six-coordinate Mn^II^ complex with Mn(ClO_4_)_2_·6H_2_O in which the Mn lies on a twofold axis. The Mn^II^ ion is significantly distorted from regular octahedral geometry as shown by the deviations of the angles at Mn from 90° (*cis*) and 180° (*trans*). The *cis* N(py)—Mn—N(py) angles are larger (100.28 (12)°) due to bulky group crowding while the *N*(amine)—Mn—*N*(amine) angles are smaller than 90°. The Mn—N bond lengths show Mn—*N*(amine) (2.365 (3) Å) > Mn—N(py) (2.200 (3) and 2.261 (3) Å). In the related structure of [Mn(bpa)_2_]^2+^ (bpa = bis(pyridin-2-ylmethyl)amine) with C_2_ symmetry (Glerup *et al.*, (1992) the observed order is Mn–N(pyr) > Mn–*N*(amine), whereas in crystals showing both C_2_ and C_i_ isomers in the same unit cell the reverse order is observed; Mn—*N*(amine) > Mn—N(py) (Gultneh *et al.*, 2006).

The perchlorate anion is disordered and was constrained to be tetrahedral with two orientations of occupancies of 0.768 (4) and 0.232 (4). The 6-methylpyridine ring was also disordered with two orientations having occupancies of 0.508 (15) and 0.492 (15).

The packing arrangement is stabilized by weak C—H···O interactions between cations and anions which link the moieties into a zigzag ribbon in the [101] direction.

### 2. Experimental

The ligand *L* was synthesized by the reaction of bis(pyridin-2-ylmethyl)amine (bpa) (Romary *et al.*, 1967) as follows: 2.2 g (11.11 mmol) was dissolved in 15.0 ml of distilled water at 0°C, and 2-methylbenzyl bromide (2.06 g, 11.1 mmol) was added. The mixture was stirred at 0°C for one hour and 0.44 g of NaOH dissolved in 10.0 ml of distilled water was added to it. The mixture was stirred for three days and extracted with methylene chloride (3x40 ml). The extracts were combined and dried over anhydrous MgSO_4_ overnight. The MgSO_4_ was filtered off and the filtrate concentrated to give 3.11 g (85% yield) of yellow oil. ^1^ H NMR CDCl_3_—TMS) (p.p.m.) 8.50 [d (H6A/B) 2H]; 7.51 [m, (H3A/B, H4A/B, H5A/B) 6H]; 7.10 [m, (C3C, C4C, C5C, C6C) 4H]; 3.81[s, (C1A/B) 4H]; 3.68 [s, (C8C) 2H]; 2.25[s, (C1C) 3H].

To 3.2 g (10.56 mmol) of *L* dissolved in 15 ml of methanol was added 1.91 g (5.28 mmol) of Mn(ClO_4_)_2_·6H_2_O under an argon atmosphere using Schlenk apparatus and the mixture was stirred overnight. To the colorless solution was added 70 ml of ether which resulted in a colorless precipitate. This was filtered under an argon atmosphere to give 3.3 g (74% yield) of a white powder which was recrystallized by layering ether on a solution of the complex in acetonitrile. IR (mineral oil) 2002,1600, 1570, 1461, 1445, 1391, 1297, 1192, 1078, 1008, 969, 869, 760, 730, 611, 507 cm^-1^.

### 3. Refinement

H atoms were placed in geometrically idealized positions with a C—H distances of 0.93 and 0.97 Å *U*_iso_(H) = 1.2*U*_eq_(C) and 0.96 Å for CH_3_ [*U*_iso_(H) = 1.5*U*_eq_(C)]. Both the perchlorate anion and one of the phenyl rings of the cation were disordered. For the anion this was modeled as an idealized tetrahedron with two orientations having occupancies of 0.737 (5) and 0.263 (5). The 6-methylpyridine ring was disordered with two orientations having occupancies of 0.536 (16) and 0.464 (16).

### Crystal data

**Table:**

[Mn(C_20_H_21_N_3_)_2_](ClO_4_)_2_	*F*(000) = 1788
*M**_r_* = 860.63	*D*_x_ = 1.388 Mg m^−^^3^
Monoclinic, *C*2/*c*	Mo *K*α radiation, λ = 0.71073 Å
*a* = 23.162 (3) Å	Cell parameters from 45 reflections
*b* = 10.4755 (11) Å	θ = 5.0–12.5°
*c* = 19.391 (2) Å	µ = 0.51 mm^−^^1^
β = 118.896 (8)°	*T* = 293 K
*V* = 4119.1 (9) Å^3^	Plate, colorless
*Z* = 4	0.42 × 0.37 × 0.18 mm

### Data collection

**Table:**

Bruker P4 diffractometer	*R*_int_ = 0.021
ω scans	θ_max_ = 27.5°, θ_min_ = 2.2°
Absorption correction: empirical (using intensity measurements) (*XEMP*; Siemens, 1989)	*h* = 0→30
*T*_min_ = 0.72, *T*_max_ = 0.92	*k* = −13→0
4863 measured reflections	*l* = −25→22
4750 independent reflections	3 standard reflections every 97 reflections
3075 reflections with *I* > 2σ(*I*)	intensity decay: none

### Refinement

**Table:**

Refinement on *F*^2^	Primary atom site location: structure-invariant direct methods
Least-squares matrix: full	Secondary atom site location: difference Fourier map
*R*[*F*^2^ > 2σ(*F*^2^)] = 0.065	Hydrogen site location: inferred from neighbouring sites
*wR*(*F*^2^) = 0.193	H-atom parameters constrained
*S* = 1.03	*w* = 1/[σ^2^(*F*_o_^2^) + (0.0957*P*)^2^ + 3.680*P*] where *P* = (*F*_o_^2^ + 2*F*_c_^2^)/3
4750 reflections	(Δ/σ)_max_ < 0.001
299 parameters	Δρ_max_ = 0.67 e Å^−^^3^
86 restraints	Δρ_min_ = −0.44 e Å^−^^3^

### Special details

**Table:**

Geometry. All e.s.d.'s (except the e.s.d. in the dihedral angle between two l.s. planes) are estimated using the full covariance matrix. The cell e.s.d.'s are taken into account individually in the estimation of e.s.d.'s in distances, angles and torsion angles; correlations between e.s.d.'s in cell parameters are only used when they are defined by crystal symmetry. An approximate (isotropic) treatment of cell e.s.d.'s is used for estimating e.s.d.'s involving l.s. planes.

### Fractional atomic coordinates and isotropic or equivalent isotropic displacement parameters (Å^2^)

**Table:**

	*x*	*y*	*z*	*U*_iso_*/*U*_eq_	Occ. (<1)
Mn1	0.5000	0.63686 (7)	0.7500	0.0433 (2)	
N1	0.48410 (13)	0.5017 (3)	0.64439 (16)	0.0464 (6)	
N1A	0.45745 (14)	0.7660 (3)	0.64777 (16)	0.0494 (7)	
N1B	0.60038 (14)	0.6010 (3)	0.76090 (17)	0.0514 (7)	
C1A	0.48177 (18)	0.5908 (4)	0.5841 (2)	0.0516 (8)	
H1AA	0.4592	0.5495	0.5331	0.062*	
H1AB	0.5265	0.6087	0.5951	0.062*	
C2A	0.44771 (16)	0.7155 (3)	0.57984 (19)	0.0491 (8)	
C3A	0.4120 (2)	0.7793 (4)	0.5091 (2)	0.0662 (11)	
H3AA	0.4051	0.7424	0.4621	0.079*	
C4A	0.3868 (2)	0.8982 (5)	0.5094 (3)	0.0752 (12)	
H4AA	0.3632	0.9427	0.4625	0.090*	
C5A	0.3967 (2)	0.9508 (4)	0.5788 (3)	0.0699 (11)	
H5AA	0.3795	1.0307	0.5798	0.084*	
C6A	0.4326 (2)	0.8826 (4)	0.6471 (2)	0.0593 (9)	
H6AA	0.4399	0.9183	0.6946	0.071*	
C1B	0.54401 (18)	0.4219 (4)	0.6743 (2)	0.0567 (9)	
H1BA	0.5474	0.3871	0.6301	0.068*	
H1BB	0.5401	0.3510	0.7040	0.068*	
C2B	0.60584 (17)	0.4959 (4)	0.7263 (2)	0.0544 (9)	
C3B	0.6657 (2)	0.4520 (5)	0.7378 (3)	0.0777 (13)	
H3BA	0.6684	0.3778	0.7132	0.093*	
C4B	0.7221 (2)	0.5204 (5)	0.7869 (3)	0.0892 (15)	
H4BA	0.7634	0.4915	0.7968	0.107*	
C5B	0.7161 (2)	0.6304 (5)	0.8202 (3)	0.0777 (13)	
H5BA	0.7531	0.6788	0.8525	0.093*	
C6B	0.65518 (18)	0.6682 (4)	0.8056 (2)	0.0614 (10)	
H6BA	0.6513	0.7444	0.8277	0.074*	
C1C	0.2892 (19)	0.497 (4)	0.478 (2)	0.111 (9)	0.508 (15)
H1CA	0.2539	0.5172	0.4267	0.166*	0.508 (15)
H1CB	0.3152	0.5719	0.5014	0.166*	0.508 (15)
H1CC	0.2713	0.4667	0.5106	0.166*	0.508 (15)
C2C	0.3326 (8)	0.393 (2)	0.4713 (11)	0.064 (3)	0.508 (15)
C3C	0.3123 (8)	0.334 (3)	0.3998 (12)	0.081 (4)	0.508 (15)
H3CA	0.2703	0.3510	0.3584	0.097*	0.508 (15)
C4C	0.3534 (15)	0.251 (3)	0.3883 (12)	0.076 (5)	0.508 (15)
H4CA	0.3454	0.2299	0.3378	0.092*	0.508 (15)
C5C	0.4054 (8)	0.200 (2)	0.4526 (13)	0.065 (3)	0.508 (15)
H5CA	0.4274	0.1298	0.4472	0.078*	0.508 (15)
C6C	0.4260 (7)	0.253 (2)	0.5268 (11)	0.054 (2)	0.508 (15)
H6CA	0.4635	0.2217	0.5702	0.064*	0.508 (15)
C7C	0.3917 (7)	0.352 (2)	0.5365 (10)	0.048 (3)	0.508 (15)
C8C	0.419 (3)	0.422 (7)	0.615 (2)	0.056 (6)	0.508 (15)
H8CA	0.4270	0.3585	0.6554	0.067*	0.508 (15)
H8CB	0.3852	0.4787	0.6127	0.067*	0.508 (15)
C1CA	0.3018 (19)	0.497 (5)	0.465 (2)	0.111 (9)	0.492 (15)
H1CD	0.2613	0.4931	0.4166	0.166*	0.492 (15)
H1CE	0.3217	0.5797	0.4706	0.166*	0.492 (15)
H1CF	0.2929	0.4840	0.5084	0.166*	0.492 (15)
C2CA	0.3492 (8)	0.393 (2)	0.4668 (12)	0.064 (3)	0.492 (15)
C3CA	0.3323 (9)	0.324 (3)	0.3997 (12)	0.081 (4)	0.492 (15)
H3CB	0.2963	0.3503	0.3527	0.097*	0.492 (15)
C4CA	0.3672 (16)	0.217 (3)	0.3998 (13)	0.076 (5)	0.492 (15)
H4CB	0.3501	0.1608	0.3571	0.092*	0.492 (15)
C5CA	0.4269 (8)	0.194 (2)	0.4635 (14)	0.065 (3)	0.492 (15)
H5CB	0.4558	0.1361	0.4604	0.078*	0.492 (15)
C6CA	0.4447 (7)	0.260 (2)	0.5337 (12)	0.054 (2)	0.492 (15)
H6CB	0.4823	0.2354	0.5795	0.064*	0.492 (15)
C7CA	0.4071 (8)	0.360 (2)	0.5357 (11)	0.048 (3)	0.492 (15)
C8CA	0.425 (3)	0.426 (8)	0.614 (2)	0.056 (6)	0.492 (15)
H8CC	0.3890	0.4802	0.6068	0.067*	0.492 (15)
H8CD	0.4311	0.3614	0.6525	0.067*	0.492 (15)
Cl1	0.61415 (5)	0.08236 (10)	0.72361 (6)	0.0693 (3)	
O1	0.63792 (17)	0.1512 (3)	0.68050 (19)	0.0808 (12)	0.768 (4)
O2	0.65812 (17)	−0.0170 (3)	0.7645 (2)	0.0922 (15)	0.768 (4)
O3	0.55217 (15)	0.0312 (4)	0.6721 (2)	0.166 (3)	0.768 (4)
O4	0.6088 (3)	0.1638 (3)	0.7776 (2)	0.147 (2)	0.768 (4)
O1A	0.5534 (3)	0.1107 (10)	0.7195 (6)	0.0808 (12)	0.232 (4)
O2A	0.6241 (5)	−0.0500 (3)	0.7298 (7)	0.0922 (15)	0.232 (4)
O3A	0.6141 (5)	0.1268 (11)	0.6554 (4)	0.166 (3)	0.232 (4)
O4A	0.6649 (4)	0.1423 (10)	0.7898 (4)	0.147 (2)	0.232 (4)

### Atomic displacement parameters (Å^2^)

**Table:**

	*U*^11^	*U*^22^	*U*^33^	*U*^12^	*U*^13^	*U*^23^
Mn1	0.0452 (4)	0.0479 (4)	0.0347 (3)	0.000	0.0175 (3)	0.000
N1	0.0470 (15)	0.0474 (16)	0.0440 (14)	−0.0036 (12)	0.0214 (12)	−0.0047 (12)
N1A	0.0570 (17)	0.0508 (17)	0.0400 (14)	0.0020 (14)	0.0232 (13)	0.0021 (12)
N1B	0.0487 (16)	0.0544 (18)	0.0507 (16)	−0.0013 (13)	0.0238 (13)	−0.0028 (13)
C1A	0.057 (2)	0.057 (2)	0.0428 (17)	−0.0020 (16)	0.0256 (16)	−0.0052 (15)
C2A	0.0493 (18)	0.056 (2)	0.0390 (16)	−0.0032 (16)	0.0191 (14)	0.0008 (15)
C3A	0.070 (2)	0.076 (3)	0.0411 (18)	0.002 (2)	0.0171 (17)	0.0087 (19)
C4A	0.073 (3)	0.078 (3)	0.058 (2)	0.007 (2)	0.018 (2)	0.023 (2)
C5A	0.071 (3)	0.057 (2)	0.072 (3)	0.008 (2)	0.026 (2)	0.012 (2)
C6A	0.071 (2)	0.050 (2)	0.057 (2)	0.0049 (18)	0.0307 (19)	0.0025 (17)
C1B	0.060 (2)	0.050 (2)	0.059 (2)	0.0060 (17)	0.0270 (18)	−0.0027 (17)
C2B	0.0505 (19)	0.052 (2)	0.060 (2)	0.0020 (16)	0.0262 (17)	0.0020 (17)
C3B	0.062 (3)	0.070 (3)	0.101 (3)	0.009 (2)	0.039 (2)	−0.008 (3)
C4B	0.053 (3)	0.086 (3)	0.123 (4)	0.005 (2)	0.038 (3)	−0.004 (3)
C5B	0.048 (2)	0.084 (3)	0.087 (3)	−0.007 (2)	0.021 (2)	−0.005 (3)
C6B	0.054 (2)	0.062 (2)	0.062 (2)	−0.0085 (18)	0.0227 (18)	−0.0058 (19)
C1C	0.056 (12)	0.133 (6)	0.098 (11)	0.008 (7)	0.002 (8)	−0.028 (7)
C2C	0.036 (7)	0.087 (3)	0.066 (3)	−0.013 (5)	0.022 (4)	−0.015 (3)
C3C	0.046 (9)	0.111 (6)	0.062 (3)	−0.017 (8)	0.007 (6)	−0.014 (3)
C4C	0.101 (12)	0.064 (14)	0.057 (5)	−0.025 (8)	0.034 (6)	−0.011 (7)
C5C	0.048 (9)	0.072 (4)	0.082 (6)	−0.028 (7)	0.039 (8)	−0.030 (4)
C6C	0.035 (7)	0.056 (3)	0.070 (4)	−0.022 (6)	0.026 (5)	−0.014 (3)
C7C	0.034 (6)	0.061 (3)	0.053 (2)	−0.025 (5)	0.025 (4)	−0.011 (2)
C8C	0.055 (9)	0.061 (4)	0.052 (2)	−0.011 (6)	0.026 (3)	−0.015 (3)
C1CA	0.056 (12)	0.133 (6)	0.098 (11)	0.008 (7)	0.002 (8)	−0.028 (7)
C2CA	0.036 (7)	0.087 (3)	0.066 (3)	−0.013 (5)	0.022 (4)	−0.015 (3)
C3CA	0.046 (9)	0.111 (6)	0.062 (3)	−0.017 (8)	0.007 (6)	−0.014 (3)
C4CA	0.101 (12)	0.064 (14)	0.057 (5)	−0.025 (8)	0.034 (6)	−0.011 (7)
C5CA	0.048 (9)	0.072 (4)	0.082 (6)	−0.028 (7)	0.039 (8)	−0.030 (4)
C6CA	0.035 (7)	0.056 (3)	0.070 (4)	−0.022 (6)	0.026 (5)	−0.014 (3)
C7CA	0.034 (6)	0.061 (3)	0.053 (2)	−0.025 (5)	0.025 (4)	−0.011 (2)
C8CA	0.055 (9)	0.061 (4)	0.052 (2)	−0.011 (6)	0.026 (3)	−0.015 (3)
Cl1	0.0697 (7)	0.0699 (7)	0.0733 (7)	0.0084 (5)	0.0386 (5)	0.0158 (5)
O1	0.112 (3)	0.072 (2)	0.083 (2)	0.006 (2)	0.066 (2)	0.0191 (19)
O2	0.101 (3)	0.078 (3)	0.098 (3)	0.022 (2)	0.048 (3)	0.028 (2)
O3	0.110 (4)	0.196 (6)	0.154 (5)	−0.040 (4)	0.033 (3)	0.002 (4)
O4	0.232 (6)	0.129 (4)	0.126 (4)	0.051 (4)	0.125 (4)	0.009 (3)
O1A	0.112 (3)	0.072 (2)	0.083 (2)	0.006 (2)	0.066 (2)	0.0191 (19)
O2A	0.101 (3)	0.078 (3)	0.098 (3)	0.022 (2)	0.048 (3)	0.028 (2)
O3A	0.110 (4)	0.196 (6)	0.154 (5)	−0.040 (4)	0.033 (3)	0.002 (4)
O4A	0.232 (6)	0.129 (4)	0.126 (4)	0.051 (4)	0.125 (4)	0.009 (3)

### Geometric parameters (Å, º)

**Table:**

Mn1—N1A^i^	2.201 (3)	C1C—H1CA	0.9600
Mn1—N1A	2.201 (3)	C1C—H1CB	0.9600
Mn1—N1B	2.262 (3)	C1C—H1CC	0.9600
Mn1—N1B^i^	2.262 (3)	C2C—C3C	1.376 (12)
Mn1—N1^i^	2.367 (3)	C2C—C7C	1.408 (11)
Mn1—N1	2.367 (3)	C3C—C4C	1.390 (15)
N1—C8CA	1.43 (9)	C3C—H3CA	0.9300
N1—C1A	1.476 (4)	C4C—C5C	1.354 (14)
N1—C1B	1.478 (4)	C4C—H4CA	0.9300
N1—C8C	1.57 (9)	C5C—C6C	1.396 (11)
N1A—C2A	1.334 (4)	C5C—H5CA	0.9300
N1A—C6A	1.347 (5)	C6C—C7C	1.373 (11)
N1B—C2B	1.327 (5)	C6C—H6CA	0.9300
N1B—C6B	1.340 (5)	C7C—C8C	1.526 (12)
C1A—C2A	1.507 (5)	C8C—H8CA	0.9700
C1A—H1AA	0.9700	C8C—H8CB	0.9700
C1A—H1AB	0.9700	C1CA—C2CA	1.536 (12)
C2A—C3A	1.384 (5)	C1CA—H1CD	0.9600
C3A—C4A	1.376 (6)	C1CA—H1CE	0.9600
C3A—H3AA	0.9300	C1CA—H1CF	0.9600
C4A—C5A	1.367 (6)	C2CA—C3CA	1.371 (12)
C4A—H4AA	0.9300	C2CA—C7CA	1.406 (11)
C5A—C6A	1.375 (5)	C3CA—C4CA	1.388 (15)
C5A—H5AA	0.9300	C3CA—H3CB	0.9300
C6A—H6AA	0.9300	C4CA—C5CA	1.357 (14)
C1B—C2B	1.507 (5)	C4CA—H4CB	0.9300
C1B—H1BA	0.9700	C5CA—C6CA	1.397 (11)
C1B—H1BB	0.9700	C5CA—H5CB	0.9300
C2B—C3B	1.373 (5)	C6CA—C7CA	1.376 (11)
C3B—C4B	1.387 (6)	C6CA—H6CB	0.9300
C3B—H3BA	0.9300	C7CA—C8CA	1.527 (12)
C4B—C5B	1.361 (7)	C8CA—H8CC	0.9700
C4B—H4BA	0.9300	C8CA—H8CD	0.9700
C5B—C6B	1.358 (6)	Cl1—O2A	1.401 (2)
C5B—H5BA	0.9300	Cl1—O3A	1.401 (2)
C6B—H6BA	0.9300	Cl1—O4A	1.402 (2)
C1C—C2C	1.528 (12)	Cl1—O1A	1.402 (2)
			
N1A^i^—Mn1—N1A	104.11 (15)	N1B—C6B—C5B	123.1 (4)
N1A^i^—Mn1—N1B	91.45 (11)	N1B—C6B—H6BA	118.5
N1A—Mn1—N1B	100.32 (11)	C5B—C6B—H6BA	118.5
N1A^i^—Mn1—N1B^i^	100.32 (11)	C2C—C1C—H1CA	109.5
N1A—Mn1—N1B^i^	91.44 (11)	C2C—C1C—H1CB	109.5
N1B—Mn1—N1B^i^	160.87 (16)	H1CA—C1C—H1CB	109.5
N1A^i^—Mn1—N1^i^	76.92 (10)	C2C—C1C—H1CC	109.5
N1A—Mn1—N1^i^	164.06 (10)	H1CA—C1C—H1CC	109.5
N1B—Mn1—N1^i^	95.54 (10)	H1CB—C1C—H1CC	109.5
N1B^i^—Mn1—N1^i^	72.83 (10)	C3C—C2C—C7C	118.7 (11)
N1A^i^—Mn1—N1	164.06 (10)	C3C—C2C—C1C	119.1 (13)
N1A—Mn1—N1	76.92 (10)	C7C—C2C—C1C	122.2 (13)
N1B—Mn1—N1	72.83 (10)	C2C—C3C—C4C	121.1 (12)
N1B^i^—Mn1—N1	95.54 (10)	C2C—C3C—H3CA	119.4
N1^i^—Mn1—N1	106.56 (14)	C4C—C3C—H3CA	119.4
C8CA—N1—C1A	111 (3)	C5C—C4C—C3C	118.3 (16)
C8CA—N1—C1B	112 (2)	C5C—C4C—H4CA	120.9
C1A—N1—C1B	109.7 (3)	C3C—C4C—H4CA	120.9
C1A—N1—C8C	113 (2)	C4C—C5C—C6C	120.1 (13)
C1B—N1—C8C	113.2 (18)	C4C—C5C—H5CA	120.0
C8CA—N1—Mn1	114.1 (17)	C6C—C5C—H5CA	120.0
C1A—N1—Mn1	103.7 (2)	C7C—C6C—C5C	120.7 (12)
C1B—N1—Mn1	105.9 (2)	C7C—C6C—H6CA	119.6
C8C—N1—Mn1	110.6 (14)	C5C—C6C—H6CA	119.6
C2A—N1A—C6A	118.9 (3)	C6C—C7C—C2C	118.8 (11)
C2A—N1A—Mn1	115.7 (2)	C6C—C7C—C8C	120.4 (13)
C6A—N1A—Mn1	124.7 (2)	C2C—C7C—C8C	120.6 (14)
C2B—N1B—C6B	118.1 (3)	C7C—C8C—N1	119 (5)
C2B—N1B—Mn1	115.8 (2)	C7C—C8C—H8CA	107.7
C6B—N1B—Mn1	125.6 (3)	N1—C8C—H8CA	107.7
N1—C1A—C2A	114.2 (3)	C7C—C8C—H8CB	107.7
N1—C1A—H1AA	108.7	N1—C8C—H8CB	107.7
C2A—C1A—H1AA	108.7	H8CA—C8C—H8CB	107.1
N1—C1A—H1AB	108.7	C2CA—C1CA—H1CD	109.5
C2A—C1A—H1AB	108.7	C2CA—C1CA—H1CE	109.5
H1AA—C1A—H1AB	107.6	H1CD—C1CA—H1CE	109.5
N1A—C2A—C3A	121.4 (4)	C2CA—C1CA—H1CF	109.5
N1A—C2A—C1A	117.1 (3)	H1CD—C1CA—H1CF	109.5
C3A—C2A—C1A	121.3 (3)	H1CE—C1CA—H1CF	109.5
C4A—C3A—C2A	119.0 (4)	C3CA—C2CA—C7CA	118.3 (12)
C4A—C3A—H3AA	120.5	C3CA—C2CA—C1CA	119.0 (14)
C2A—C3A—H3AA	120.5	C7CA—C2CA—C1CA	122.6 (14)
C5A—C4A—C3A	120.0 (4)	C2CA—C3CA—C4CA	122.0 (14)
C5A—C4A—H4AA	120.0	C2CA—C3CA—H3CB	119.0
C3A—C4A—H4AA	120.0	C4CA—C3CA—H3CB	119.0
C4A—C5A—C6A	118.3 (4)	C5CA—C4CA—C3CA	118.5 (16)
C4A—C5A—H5AA	120.9	C5CA—C4CA—H4CB	120.7
C6A—C5A—H5AA	120.9	C3CA—C4CA—H4CB	120.7
N1A—C6A—C5A	122.5 (4)	C4CA—C5CA—C6CA	119.6 (13)
N1A—C6A—H6AA	118.8	C4CA—C5CA—H5CB	120.2
C5A—C6A—H6AA	118.8	C6CA—C5CA—H5CB	120.2
N1—C1B—C2B	112.3 (3)	C7CA—C6CA—C5CA	120.6 (12)
N1—C1B—H1BA	109.1	C7CA—C6CA—H6CB	119.7
C2B—C1B—H1BA	109.1	C5CA—C6CA—H6CB	119.7
N1—C1B—H1BB	109.1	C6CA—C7CA—C2CA	119.3 (11)
C2B—C1B—H1BB	109.1	C6CA—C7CA—C8CA	119.8 (15)
H1BA—C1B—H1BB	107.9	C2CA—C7CA—C8CA	120.7 (14)
N1B—C2B—C3B	122.1 (4)	N1—C8CA—C7CA	114 (6)
N1B—C2B—C1B	118.2 (3)	N1—C8CA—H8CC	108.6
C3B—C2B—C1B	119.7 (4)	C7CA—C8CA—H8CC	108.6
C2B—C3B—C4B	118.8 (4)	N1—C8CA—H8CD	108.6
C2B—C3B—H3BA	120.6	C7CA—C8CA—H8CD	108.6
C4B—C3B—H3BA	120.6	H8CC—C8CA—H8CD	109.0
C5B—C4B—C3B	119.0 (4)	O2A—Cl1—O3A	109.53 (9)
C5B—C4B—H4BA	120.5	O2A—Cl1—O4A	109.54 (9)
C3B—C4B—H4BA	120.5	O3A—Cl1—O4A	109.50 (9)
C6B—C5B—C4B	118.8 (4)	O2A—Cl1—O1A	109.45 (9)
C6B—C5B—H5BA	120.6	O3A—Cl1—O1A	109.42 (9)
C4B—C5B—H5BA	120.6	O4A—Cl1—O1A	109.38 (9)
			
C8CA—N1—C1A—C2A	86.2 (15)	C4B—C5B—C6B—N1B	1.3 (7)
C1B—N1—C1A—C2A	−149.7 (3)	C7C—C2C—C3C—C4C	−10 (4)
C8C—N1—C1A—C2A	83.0 (13)	C1C—C2C—C3C—C4C	172 (4)
Mn1—N1—C1A—C2A	−36.8 (3)	C2C—C3C—C4C—C5C	18 (5)
C6A—N1A—C2A—C3A	−0.9 (5)	C3C—C4C—C5C—C6C	−15 (5)
Mn1—N1A—C2A—C3A	169.7 (3)	C4C—C5C—C6C—C7C	4 (4)
C6A—N1A—C2A—C1A	174.3 (3)	C5C—C6C—C7C—C2C	4 (3)
Mn1—N1A—C2A—C1A	−15.1 (4)	C5C—C6C—C7C—C8C	−172 (5)
N1—C1A—C2A—N1A	37.9 (4)	C3C—C2C—C7C—C6C	−2 (3)
N1—C1A—C2A—C3A	−146.9 (3)	C1C—C2C—C7C—C6C	176 (3)
N1A—C2A—C3A—C4A	0.9 (6)	C3C—C2C—C7C—C8C	175 (5)
C1A—C2A—C3A—C4A	−174.2 (4)	C1C—C2C—C7C—C8C	−7 (6)
C2A—C3A—C4A—C5A	−0.8 (7)	C6C—C7C—C8C—N1	66 (6)
C3A—C4A—C5A—C6A	0.7 (7)	C2C—C7C—C8C—N1	−110 (3)
C2A—N1A—C6A—C5A	0.9 (6)	C1A—N1—C8C—C7C	50 (4)
Mn1—N1A—C6A—C5A	−168.8 (3)	C1B—N1—C8C—C7C	−75 (4)
C4A—C5A—C6A—N1A	−0.8 (7)	Mn1—N1—C8C—C7C	166 (3)
C8CA—N1—C1B—C2B	−164 (3)	C7CA—C2CA—C3CA—C4CA	6 (4)
C1A—N1—C1B—C2B	72.3 (4)	C1CA—C2CA—C3CA—C4CA	−170 (4)
C8C—N1—C1B—C2B	−160 (2)	C2CA—C3CA—C4CA—C5CA	−14 (6)
Mn1—N1—C1B—C2B	−39.1 (3)	C3CA—C4CA—C5CA—C6CA	15 (5)
C6B—N1B—C2B—C3B	3.0 (6)	C4CA—C5CA—C6CA—C7CA	−10 (4)
Mn1—N1B—C2B—C3B	−170.1 (3)	C5CA—C6CA—C7CA—C2CA	2 (3)
C6B—N1B—C2B—C1B	−178.5 (3)	C5CA—C6CA—C7CA—C8CA	177 (5)
Mn1—N1B—C2B—C1B	8.5 (4)	C3CA—C2CA—C7CA—C6CA	0 (3)
N1—C1B—C2B—N1B	22.8 (5)	C1CA—C2CA—C7CA—C6CA	175 (3)
N1—C1B—C2B—C3B	−158.6 (4)	C3CA—C2CA—C7CA—C8CA	−175 (5)
N1B—C2B—C3B—C4B	−0.4 (7)	C1CA—C2CA—C7CA—C8CA	1 (6)
C1B—C2B—C3B—C4B	−178.9 (4)	C1A—N1—C8CA—C7CA	51 (4)
C2B—C3B—C4B—C5B	−1.8 (8)	C1B—N1—C8CA—C7CA	−72 (4)
C3B—C4B—C5B—C6B	1.3 (8)	Mn1—N1—C8CA—C7CA	168 (2)
C2B—N1B—C6B—C5B	−3.5 (6)	C6CA—C7CA—C8CA—N1	71 (6)
Mn1—N1B—C6B—C5B	168.8 (3)	C2CA—C7CA—C8CA—N1	−114 (3)

Symmetry code: (i) −*x*+1, *y*, −*z*+3/2.

### Hydrogen-bond geometry (Å, º)

**Table:**

*D*—H···*A*	*D*—H	H···*A*	*D*···*A*	*D*—H···*A*
C3*A*—H3*AA*···O3*A*^ii^	0.93	2.51	3.106 (5)	122
C6*A*—H6*AA*···Cl1^iii^	0.93	2.99	3.802 (4)	146
C6*A*—H6*AA*···O1*A*^iii^	0.93	2.57	3.420 (10)	152
C6*A*—H6*AA*···O2*A*^iii^	0.93	2.56	3.309 (12)	138
C1*B*—H1*BB*···O4	0.97	2.49	3.266 (5)	136
C1*B*—H1*BB*···O1*A*	0.97	2.54	3.356 (11)	142
C3*B*—H3*BA*···O1	0.93	2.47	3.300 (6)	149

Symmetry codes: (ii) −*x*+1, −*y*+1, −*z*+1; (iii) −*x*+1, *y*+1, −*z*+3/2.
